# Supplementary material for: Pathways for horizontal gene transfer in bacteria revealed by a global map of their plasmids
Source: Nat Commun. 2020 Jul 17;11:3602. doi: 10.1038/s41467-020-17278-2 (PMC7367871; doi:10.1038/s41467-020-17278-2)
Supplement: Supplementary file 1 — Supplementary Information [file 41467_2020_17278_MOESM1_ESM.pdf]

Supplementary Information

Pathways for Horizontal Gene Transfer in Bacteria Revealed by a Global Map of their Plasmids

Redondo-Salvo et al.

Supplementary Methods: Community identification using stochastic blockmodels (SBM)

Stochastic blockmodels are a family of algorithms employed to identify clusters or communities within graphs. SBMs combine generative models and Bayesian statistics to evaluate the statistical support of the communities observed in the problem graph[1]. This way SBMs are able to rule out graph structure arising by chance, due to the random distribution of nodes and edges. The versatility and statistical support offered by SBMs have made them popular in the fields of computer science, machine learning and statistics[2].

In this work we used graph-tool 2.29 a Python module for network manipulation and statistical analysis[3]. SBM is based on nonparametric statistical inference. SBMs are used as generative models containing communities that are detected by inferring the model parameters from the problem data. The general principle is to infer the Bayesian posterior probability of observing a particular network partition (*b*) in a certain network (*A*), given the set of parameters (*θ*):

$$P(b|A) = \frac{P(A|\theta, b)\mathrm{P}(\theta,b)}{P(A)} \qquad (1)$$

The inference procedure consists in finding the network partition b that maximizes Eq. (1)[4]. This is performed by turning probability distribution into entropies and looking at the description length of the network $\Sigma = -\ln \mathrm{P}(A|\theta,b) - \ln \mathrm{P}(\theta,b)$. Obtaining the minimum description length (MDL) is then equivalent to maximizing the posterior Bayesian probability.

In this analysis we tested four types of SBMs:

- <u>Flat SBM:</u> Using this algorithm nodes are divided into a partition, *b*, including *B* different communities. The network is generated from a *B* x *B* matrix of edge counts between groups. In the flat SBM, edges are placed randomly inside each group, so members of the same group tend to have similar degrees.

- <u>Degree-Corrected SBM (DC-SBM):</u> In many naturally occurring networks, members of the same community tend to have a heterogeneous degree

distribution. DC-SBM introduces a new set of parameters, *k*, indicating the degree distribution of the graph.

- Hierarchical SBM (HSBM): Flat SBMs present a limit on the maximum number of communities of the order $O(\sqrt{N})$, which in our case would severely limit the detection of PTUs with more than 14 members. Nested SBMs solve this problem by introducing a nested structure, were clusters are organized hierarchically according to a set of priors and hyperpriors, reaching a number of communities of $O(N/\log(N))$.

- Degree-Corrected Hierarchical SBM (DC-HSBM): Introduce a degree correction, as in DC-SBMs, and allow a nested structure as HSBMs.

Choosing the model with the minimum description length

First, we tested the four basic SBM algorithms on our problem dataset, to identify the one that:

a) Yielded the minimum description length of the partition.

b) Generated biologically-plausible communities, that is, communities in which our members have some detectable ANI score. Groups including members with no detectable DNA-DNA homology cannot be considered members of the same "molecular species".

When applied to the set of enterobacterales plasmids, results were the following:

Algorithm	**MDL**	**# of detected groups**	**# Non-biological groups**
SBM	82128.339	60	YES
DC-SBM	82391.510	58	YES
HSBM	71710.781	213	YES
DC-HSBM	72810.274	183	YES

And when applied to the entire RefSeq84 database, results were:

Algorithm	**MDL**	**# of detected groups**	**# Non-biological groups**
SBM	197592.477	102	YES
DC-SBM	195559.926	98	YES
HSBM	159837.233	534	YES
DC-HSBM	162010.212	473	YES

Avoiding biologically-implausible communities

In all cases, SBM algorithms produced communities containing plasmids that showed no detectable DNA homology between each other. These groups are, as described before, biologically implausible and should be ascribed a 0 posterior probability. In order to amend this problem, we chose the algorithm with the optimal MDL (HSBM), and introduced the following modifications:

- *Blocks are divided into their connected components.* Blocks composed by disjoint connected components are partitioned accordingly. This removes blocks formed by sets of plasmids with no DNA homology among them.

- *Blocks with less than 4 members are discarded.* Assessing the conservation of the common genomic backbone of the PTU can be performed reliably in groups with at least 4 members. Smaller PTUs are not considered, awaiting for further sequencing efforts increase the sampling of these minority groups.

These two conditions are equivalent to change the null hypothesis. HSBM assumes that, in the absence of evidence, all plasmids belong to the same structure (PTU). Our model reverses this assumption: in the absence of evidence, any two plasmids belong to different molecular species, hence assigned to different PTUs. We named this algorithm sHSBM.

Testing the algorithm in simulated networks

In order to check the performance of our modified algorithm we benchmarked it using a set of simulated networks that reproduced the structure found in the enterobacterales dataset. This was achieved by generating networks with approximately the same number of members (2,500) and distributed into 300 PTUs. PTU membership was modeled after a gamma distribution with shape parameter $k = 0.3$, and scale $\theta = 1$. These parameters were chosen because they produced the same proportion of singletons as our problem network. Intragroup edge distribution was simulated by looking at completely isolated communities in the problem graph, which according to MOB and rep distribution likely corresponded to single PTUs. Intergroup edge distribution was also modeled after experimental data. In this case, we used the distribution of ANI scores experimentally determined for the enterobacterales dataset and fitted it to an exponential distribution.

A total of 1,000 simulated networks were generated. A representation of one of these simulated networks is shown in Supplementary Figure 7.

The performance of the algorithm was then tested by looking at the normalized mutual information score (NMI) as described by Fortunato[5]. Using the default HSBM, the average NMI score of the simulated set was NMI=0.965967 and a standard deviation of std=0.00458. Using the HSBM algorithm adjusted for discarding biologically-implausible communities, sHSBM results were NMI=0.99674, std=0.00075. Results thus demonstrated that our method improved PTU recovery.

Applying sHSBM to the problem set

Once the performance of sHSBM was benchmarked, we applied it to our two problem datasets: that including plasmids from the order *Enterobacterales* (2,535 nodes) and that including all curated RefSeq84 plasmids (9,894).

The level of agreement of sHSBM and the topological PID algorithm described in the main text was checked as follows:

- For each sHSBM group, the list of plasmids belonging to that group is extracted.
- For each plasmid, we checked its corresponding PID label. That is, the group assigned by the topological PID algorithm.

- The most frequent PID label in that plasmid subset is calculated.
- The most frequent PID label is then assigned to that particular sHSBM label.
- Finally, an agreement score is generated. If the number of members with the same PID an sHSBM is identical, we assign a *perfect* score. If the number of members with the same PID and sHSBM is not identical, but higher than 90% of the most numerous, we assign a *high* score. If the number of common members is 66.6-90% we assign a *medium* score, and *low* otherwise.

The complete PTU list of the order *Enterobacterales* showing the PID / sHSBM agreement is found in Supplementary Data 4.

Code Availability

Scripts required for performing the analyses described here can be found in https://github.com/santirdnd/ptu_paper/.

The code was executed under the following dependencies:

- Python v.3.6 using ANACONDA, including NumPy, PANDAS and scikit-Learn v.022.
- Graph-tools 2.29 installed through ANACONDA.

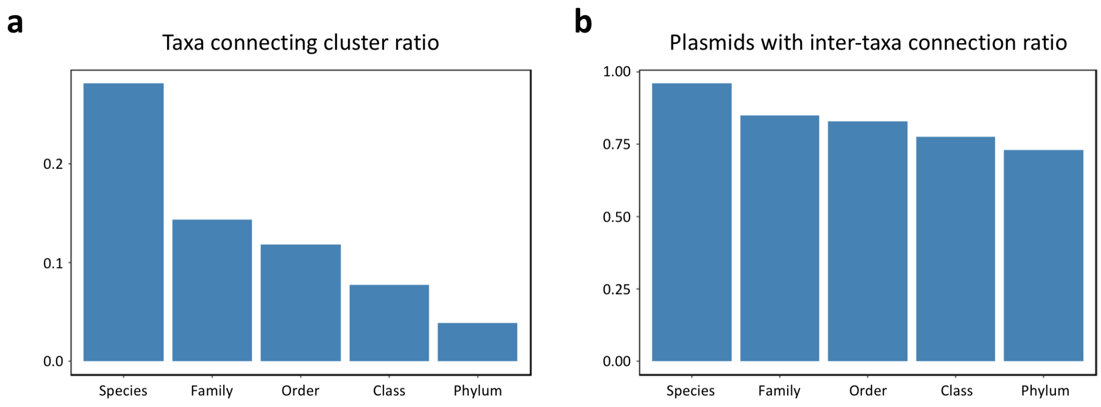

Supplementary Figure 1. Evaluation of inter-clade connectivity on plasmidome/proteome network. Inter-clade connections of the RefSeq84 AcCNET network show a decrease in the connectivity as the taxonomic level is increased. **(a)** Bar plot of the ratio of clusters connecting plasmids of different taxa, analyzed by taxonomic level. **(b)** Bar plot of the ratio of plasmids that are connected to other plasmids of different taxa, analyzed by taxonomic level.

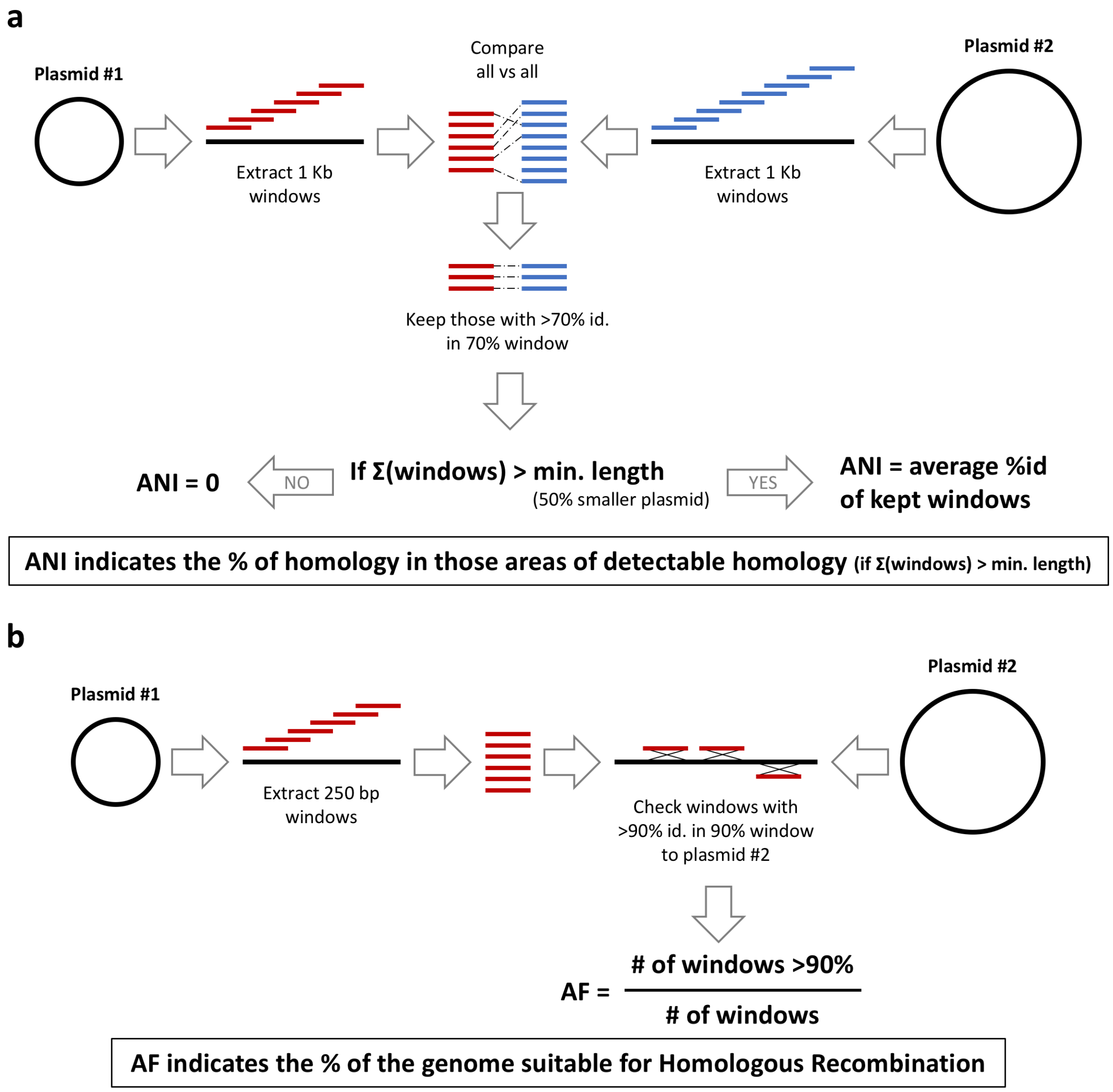

Supplementary Figure 2. Algorithms for ANI and AF calculation. (a) Graphical representation of the algorithm used for the ANI_{L50} plasmid pairwise comparison. **(b)** Graphical representation of the algorithm used to calculate the annealing fraction (AF) between 2 plasmids.

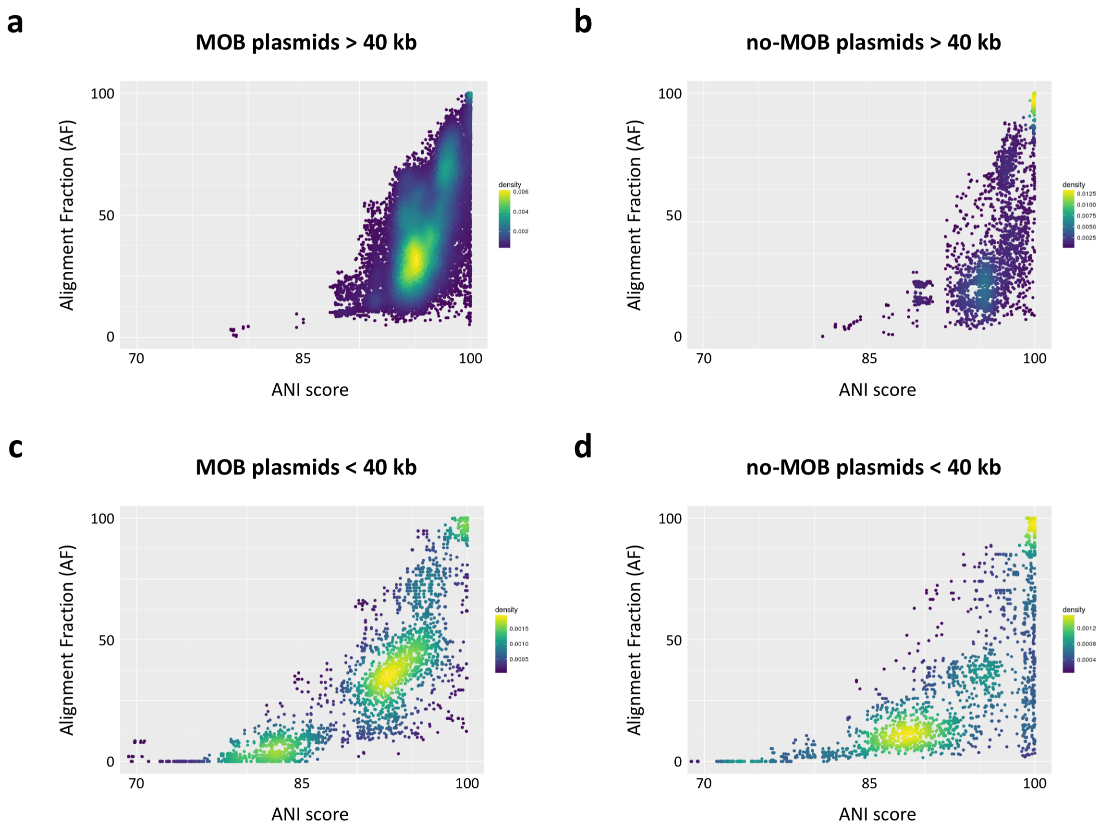

Supplementary Figure 3. AF vs ANI scatterplots factored by MOB presence and genome length (40 kb). (a) AF vs ANI scatterplot of MOB plasmids bigger than 40 kb. **(b)** AF vs ANI scatterplot of no-MOB plasmids bigger than 40 kb. **(c)** AF vs ANI scatterplot of MOB plasmids smaller than 40 kb. **(d)** AF vs ANI scatterplot of no-MOB plasmids smaller than 40 kb.

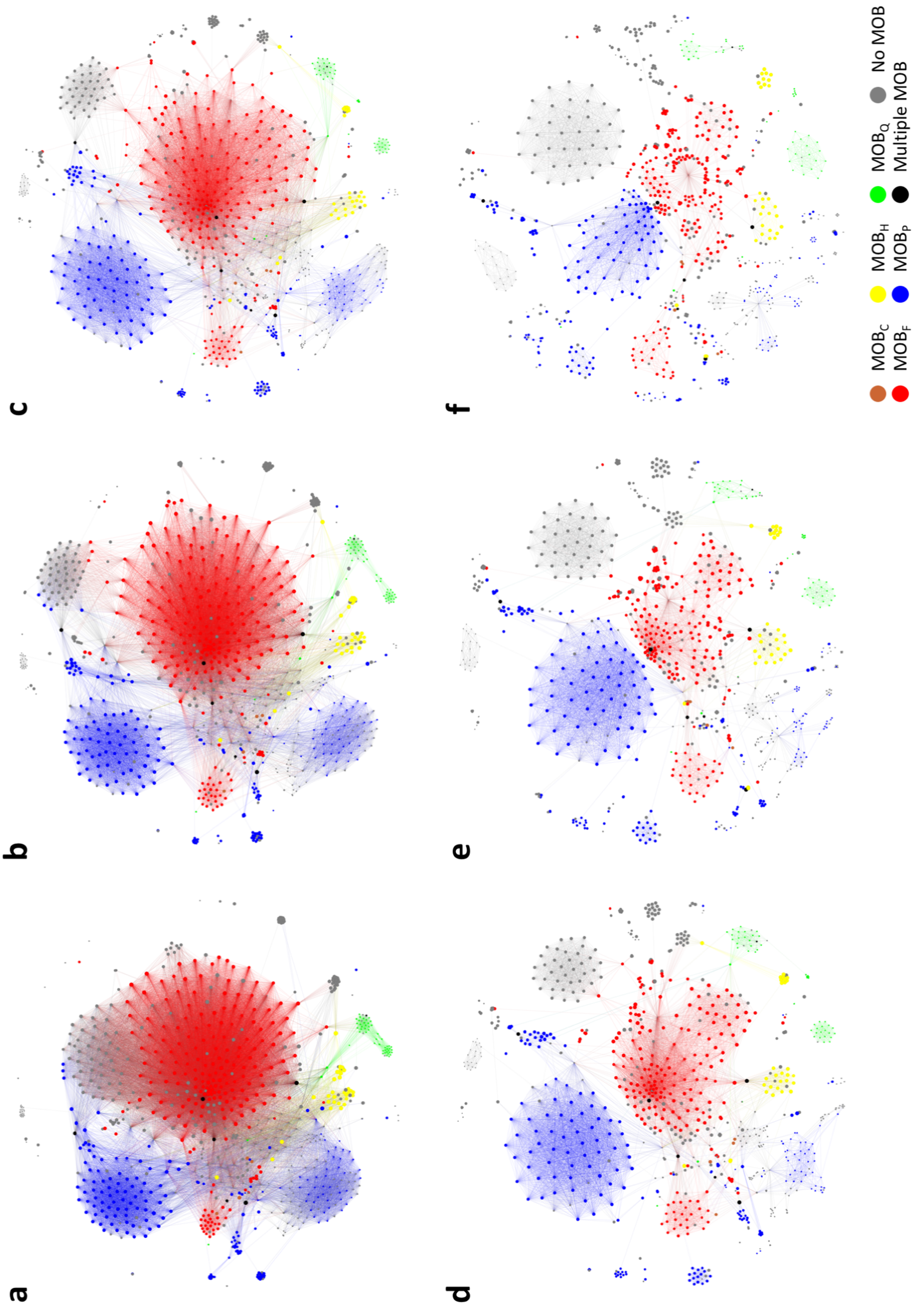

Supplementary Figure 4. Network connectivity depending on ANI thresholds. ANI networks obtained for the *E. coli* plasmidome using different minimal length criteria, with plasmids colored according to their MOB type. **(a)** ANI_{L20}, **(b)** ANI_{L30}, **(c)** ANI_{L40}, **(d)** ANI_{L50}, **(e)** ANI_{L60}, **(f)** ANI_{L70}.

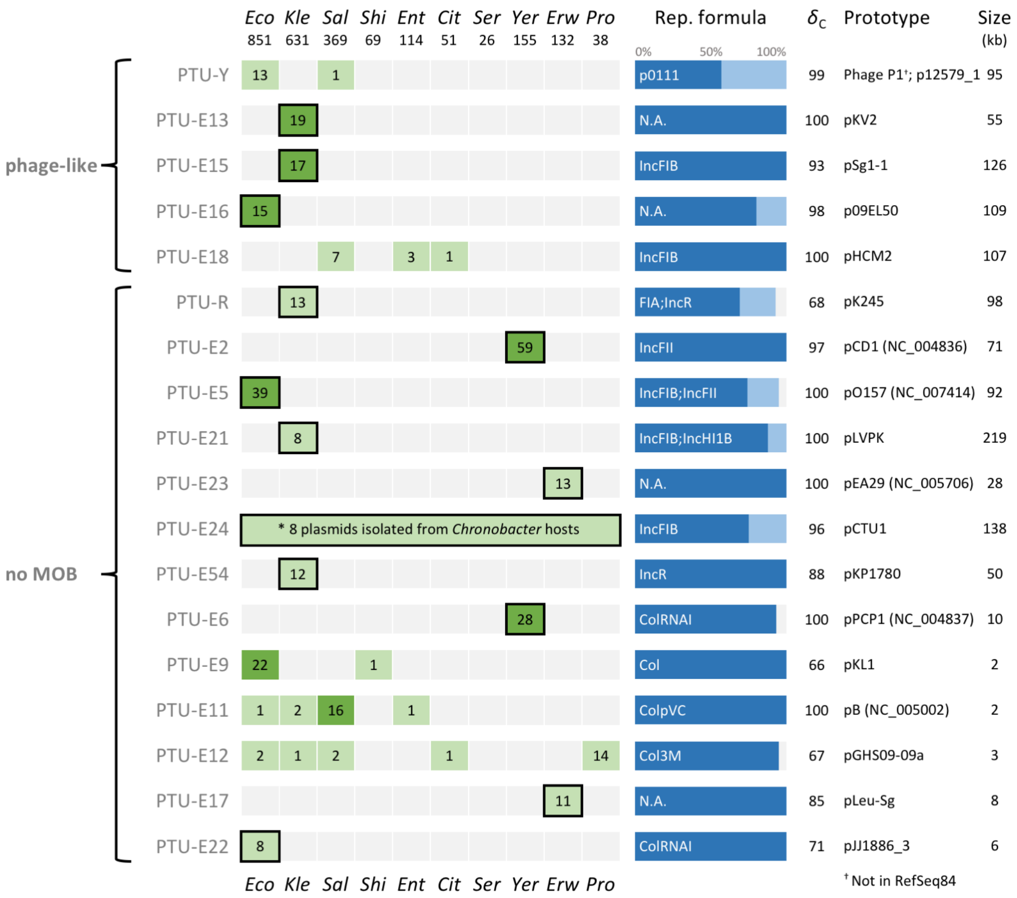

Supplementary Figure 5. Summary of the most representative non-mobilizable PTUs in the order *Enterobacterales*. Together with the conjugative and mobilizable PTUs shown in Figure 4c, they represent 69% of enterobacterales plasmids (1,770 out of 2,535). The remaining 28 PTUs not shown in this figure represent an additional 5% of the enterobacterales plasmidome. Supplementary Data 4 contains a comprehensive list of all PTUs detected in *Enterobacterales*. First columns indicate, for each particular PTU, the number of plasmids per taxon as indicated in the upper row (all representing the genus level except for the two last columns, representing the *Erwiniaceae* and *Morganellaceae* families). Dark green squares indicate high plasmid prevalence (n>15). Squares with a black border indicate PTUs with presence on only one host genus. The next column shows the distribution of the most abundant replicon formula (each bluish band represents a different replicon set with > 10 % abundance. Replicons have been annotated by PlasmidFinder 1.3 with 80% threshold). On the rightmost part of the figure, the chart shows the intra-cluster density, as a measure of cluster quality, and suggests a prototype for the PTU.

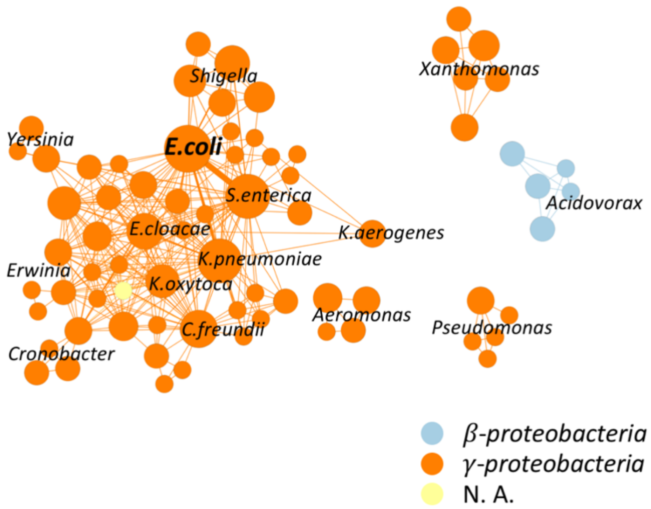

Supplementary Figure 6. Exchange communities depend on Grade V and VI PTUs. Exchange map for *Enterobacterales*, as shown in Figure 7 in the main text, when plasmids from Grade V and Grade VI are removed. A comparison between this figure and Figure 7 reveals that the exchange community disaggregates in its composing orders when Grade VI and V PTUs are not considered.

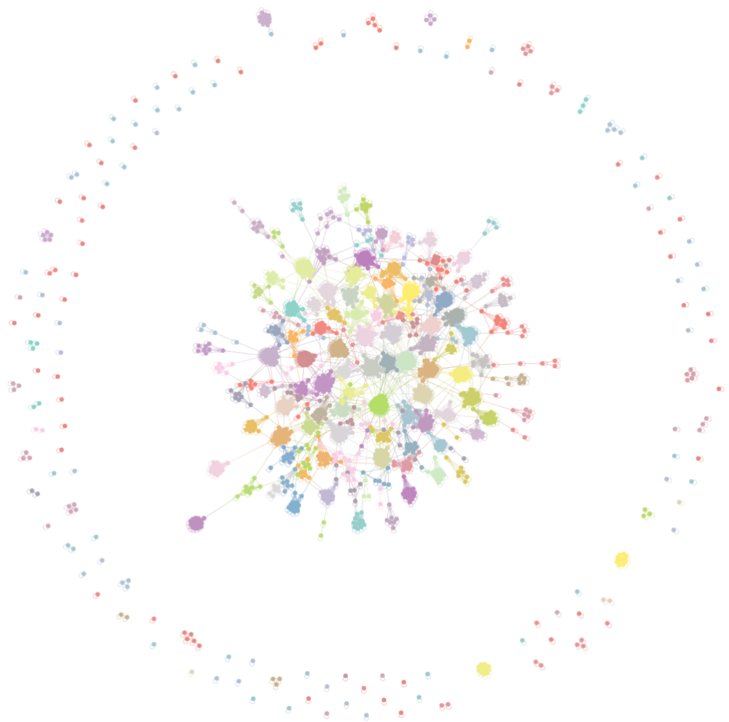

Supplementary Figure 7. A simulated network for SBM benchmarking. This network was generated using the procedure described in the Supplementary Methods section. An automatic color scheme was used to color the predicted block membership of the different nodes.

Cluster ID	*Grade*	*Descriptor (1)*	*Predicted function*	*Phylum (2)*
Cluster_421162	3139	>WP_040125287.1 IS6 family transposase	Transposase IS240 / IS26	Proteobacteria; Actinobacteria; Firmicutes; -; Cyanobacteria
Cluster_425340	998	>NP_878010.1 IS431mec transposase	Transposase IS240 / IS26	Firmicutes
Cluster_9838	862	>YP_001692940.1 transposase	Tn3 Transposase	Proteobacteria; Actinobacteria; Firmicutes; Cyanobacteria; Nitrospirae
Cluster_711816	839	>WP_015074896.1 IS66 family insertion sequence hypothetical protein	TnpB IS66	Proteobacteria
Cluster_273625	794	>WP_065284761.1 IS5/IS1182 family transposase	Transposase IS5 family	Proteobacteria
Cluster_242047	674	>WP_087760817.1 IS5/IS1182 family transposase	Transposase IS5 family	Proteobacteria; Firmicutes; Acidobacteria
Cluster_352805	629	>WP_051480104.1 TEM family class A beta-lactamase	Ampicilin resistance	Spirochaetes; Proteobacteria; Actinobacteria; Firmicutes
Cluster_413653	621	>YP_003560434.1 hypothetical protein	Adenosyl-methionine-dependent methyltransferases	Proteobacteria
Cluster_199167	617	>NP_444055.1 transposase	Transposase (IS116, IS110 and IS902 Family)	Proteobacteria; Acidobacteria
Cluster_88697	603	>WP_012555755.1 sugar ABC transporter ATP-binding protein	ABC Sugar transporter	Proteobacteria; Actinobacteria; Chloroflexi
Cluster_339622	600	>WP_088546682.1 incFII family plasmid replication initiator RepA	Plasmid Replication (IncFII RepA)	Proteobacteria
Cluster_834005	576	>WP_008324202.1 hypothetical protein	Unknown Function	Proteobacteria
Cluster_592754	548	>YP_003829028.1 plasmid SOS inhibition protein B	SOS inhibition	Proteobacteria
Cluster_371716	547	>WP_035994305.1 amino acid ABC transporter ATP-binding protein	ABC Aminoacid Transporter	Proteobacteria; Actinobacteria; Firmicutes; Deinococcus-Thermus

Supplementary Table 1. Most abundant protein clusters (connected to >5% of the plasmidome). (1) Protein with the highest overall identity to all cluster members. (2) Phyla with plasmids connected to the cluster.

Cluster ID	*Grade*	*Descriptor (1)*	*Antibiotic*	*Phylum (2)*
Cluster_352805	629	>WP_051480104.1 TEM family class A beta-lactamase	B-Lactams	Spirochaetes; Proteobacteria; Actinobacteria; -; Firmicutes
Cluster_696126	401	>YP_009163988.1 QacEdelta1 multidrug exporter	Amonium	Proteobacteria; Actinobacteria
Cluster_150559	386	>YP_003829179.1 tetracycline efflux protein	Tetracycline	Proteobacteria; Actinobacteria; Deinococcus-Thermus; -
Cluster_309903	376	>WP_063857834.1 carbapenem-hydrolyzing class A beta-lactamase BKC-1	B-Lactams	Proteobacteria
Cluster_371263	285	>YP_003517601.1 aminoglycoside/hydroxyurea antibiotic resistance kinase	Aminogylcosides	Proteobacteria; Actinobacteria
Cluster_470505	279	>YP_003108338.1 aminoglycoside N(6')-acetyltransferase	Aminogylcosides	Proteobacteria; Actinobacteria
Cluster_478668	207	>YP_006954662.1 quinolone resistance protein	Quinolone	Proteobacteria
Cluster_170254	191	>WP_095436767.1 class C beta-lactamase	B-Lactams	Proteobacteria
Cluster_343631	124	>YP_006958171.1 beta-lactamase	B-Lactams	Firmicutes
Cluster_390636	119	>WP_032495622.1 subclass B1 metallo-beta-lactamase NDM-7	B-Lactams	Proteobacteria
Cluster_345531	100	>WP_063860498.1 carbapenem-hydrolyzing class A beta-lactamase FRI-1	B-Lactams	Proteobacteria

Supplementary Table 2. Most abundant antibiotic resistance clusters (present in >1% of the plasmidome). (1) Protein with the highest overall identity to all cluster members. (2) Phyla with plasmids connected to the cluster.

Supplementary References

1. Holland, P. W., Laskey, K. B. & Leinhardt, S. Stochastic blockmodels: First steps. *Soc. Netw.* **5**, 109–137 (1983).
2. Abbe, E. Community Detection and Stochastic Block Models: Recent Developments. *J. Mach. Learn. Res.* **18**, 1–86 (2018).
3. Peixoto, T. P. The graph-tool python library. (2017) doi:10.6084/m9.figshare.1164194.v14.
4. Peixoto, T. P. Bayesian Stochastic Blockmodeling. in *Advances in Network Clustering and Blockmodeling* 289–332 (John Wiley & Sons, Ltd, 2019). doi:10.1002/9781119483298.ch11.
5. Fortunato, S. Community detection in graphs. *Phys. Rep.* **486**, 75–174 (2010).
