# Supplementary material for: Pathways for horizontal gene transfer in bacteria revealed by a global map of their plasmids
Source: Nat Commun. 2020 Jul 17;11:3602. doi: 10.1038/s41467-020-17278-2 (PMC7367871; doi:10.1038/s41467-020-17278-2)
Supplement: Supplementary file 3 — Description of Additional Supplementary Files [file 41467_2020_17278_MOESM3_ESM.pdf]

Description of Additional Supplementary Files

File name: Supplementary Data 1

Description: A table containing the protein clusters distributed across 6 or more phyla.

File name: Supplementary Data 2

Description: List of the 276 PTUs in the bacterial plasmidome.

File name: Supplementary Data 3

Description: Inter and Intracluster densities for all PTUs.

File name: Supplementary Data 4

Description: List of the 83 PTUs in the enterobacteral plasmidome defined by PID and sHSBM.

File name: Supplementary Data 5

Description: Table containing the metadata for all plasmid sequences analysed in this study.
