# Supplementary material for: Pathways for horizontal gene transfer in bacteria revealed by a global map of their plasmids
Source: Nat Commun. 2020 Jul 17;11:3602. doi: 10.1038/s41467-020-17278-2 (PMC7367871; doi:10.1038/s41467-020-17278-2)
Supplement: Supplementary file 9 — Reporting Summary [file 41467_2020_17278_MOESM9_ESM.pdf]

nature research

Corresponding author(s): Fernando de la Cruz

Last updated by author(s): Jun 15, 2020

Reporting Summary

Nature Research wishes to improve the reproducibility of the work that we publish. This form provides structure for consistency and transparency in reporting. For further information on Nature Research policies, see our Editorial Policies and the Editorial Policy Checklist.

Statistics

For all statistical analyses, confirm that the following items are present in the figure legend, table legend, main text, or Methods section.

n/a	Confirmed	
☒	☐	The exact sample size (n) for each experimental group/condition, given as a discrete number and unit of measurement
☒	☐	A statement on whether measurements were taken from distinct samples or whether the same sample was measured repeatedly
☒	☐	The statistical test(s) used AND whether they are one- or two-sided<br>*Only common tests should be described solely by name; describe more complex techniques in the Methods section.*
☒	☐	A description of all covariates tested
☒	☐	A description of any assumptions or corrections, such as tests of normality and adjustment for multiple comparisons
☒	☐	A full description of the statistical parameters including central tendency (e.g. means) or other basic estimates (e.g. regression coefficient) AND variation (e.g. standard deviation) or associated estimates of uncertainty (e.g. confidence intervals)
☒	☐	For null hypothesis testing, the test statistic (e.g. F, t, r) with confidence intervals, effect sizes, degrees of freedom and P value noted<br>*Give P values as exact values whenever suitable.*
☒	☐	For Bayesian analysis, information on the choice of priors and Markov chain Monte Carlo settings
☒	☐	For hierarchical and complex designs, identification of the appropriate level for tests and full reporting of outcomes
☒	☐	Estimates of effect sizes (e.g. Cohen's d, Pearson's r), indicating how they were calculated

Our web collection on statistics for biologists contains articles on many of the points above.

Software and code

Policy information about availability of computer code

Data collection	Plasmid sequences and metadata were downloaded from publicly available NCBI's RefSeq plasmid dataset (release 84). Data were retrieved using custom scripts deposited in https://github.com/santirdnd/PTU_paper/.
Data analysis	Home-made scripts are deposited in https://github.com/santirdnd/PTU_paper/. Other software used in data analysis:<br>- AcCNET v1.2<br>kClust (bundled with AcCNET)<br>- MOBscan (online tool)<br>- PlasmidFinder v1.3<br>- BLAST+ v2.6.0<br>- Gephi v0.9.2<br>ForceAtlas2 (bundled with Gephi)<br>- ani.rb (downloaded on Mar 16, 2017)<br>- Circos 0.69<br>- graph-tool v2.29

For manuscripts utilizing custom algorithms or software that are central to the research but not yet described in published literature, software must be made available to editors and reviewers. We strongly encourage code deposition in a community repository (e.g. GitHub). See the Nature Research guidelines for submitting code & software for further information.

Data

Policy information about availability of data

All manuscripts must include a data availability statement. This statement should provide the following information, where applicable:

- Accession codes, unique identifiers, or web links for publicly available datasets
- A list of figures that have associated raw data
- A description of any restrictions on data availability

The sequences conforming the analyzed dataset are available in the NCBI's RefSeq repository (https://ftp.ncbi.nlm.nih.gov/refseq/release/plasmid/). The accession numbers of the sequences are listed in the Supplementary Data 5. Taxonomy data was downloaded from NCBI's Taxonomy database on Nov 24, 2017. The source data underlying Figs. 1a-f, 3a-b, 4a-b and 6 are provided as a Source Data file.

Field-specific reporting

Please select the one below that is the best fit for your research. If you are not sure, read the appropriate sections before making your selection.

[x] Life sciences [ ] Behavioural & social sciences [ ] Ecological, evolutionary & environmental sciences

For a reference copy of the document with all sections, see nature.com/documents/nr-reporting-summary-flat.pdf

Life sciences study design

All studies must disclose on these points even when the disclosure is negative.

Sample size	The complete plasmid dataset of NCBI RefSeq release 84 was obtained. It comprised 10,634 bacterial and archaeal plasmids.
Data exclusions	740 sequences were eliminated from the dataset after a manual curation process. The pre-established criteria included the removal of partial plasmid DNA sequences, bacterial/archaeal chromosomes, sequences with unassignable hosts and/or PacBio internal control sequences.
Replication	SBMs are stochastical algorithms that were used to evaluate the clustering performance. Each algorithm was initialized 100 times, and that with the lower entropy was selected and further refined using 120,000 iterations. All replication attemps were successful.
Randomization	This is not relevant to our study. Our dataset was not split into different experimental groups, but subjected to different bioinformatic algorithms as a whole.
Blinding	This is not relevant to our study. Our dataset was not split into different experimental groups, but subjected to different bioinformatic algorithms as a whole.

Reporting for specific materials, systems and methods

We require information from authors about some types of materials, experimental systems and methods used in many studies. Here, indicate whether each material, system or method listed is relevant to your study. If you are not sure if a list item applies to your research, read the appropriate section before selecting a response.

Materials & experimental systems

n/a	Involved in the study	
[x]	[ ]	Antibodies
[x]	[ ]	Eukaryotic cell lines
[x]	[ ]	Palaeontology and archaeology
[x]	[ ]	Animals and other organisms
[x]	[ ]	Human research participants
[x]	[ ]	Clinical data
[x]	[ ]	Dual use research of concern

Methods

n/a	Involved in the study	
[x]	[ ]	ChIP-seq
[x]	[ ]	Flow cytometry
[x]	[ ]	MRI-based neuroimaging
